# Social Isolation, Loneliness and Health: A Descriptive Study of the Experiences of Migrant Mothers With Young Children (0–5 Years Old) at La Maison Bleue

**DOI:** 10.3389/fgwh.2022.823632

**Published:** 2022-06-24

**Authors:** Mona Lim, Andraea Van Hulst, Sarah Pisanu, Lisa Merry

**Affiliations:** ^1^Ingram School of Nursing, McGill University, Montreal, QC, Canada; ^2^La Maison Bleue, Montreal, QC, Canada; ^3^Faculty of Nursing, University of Montreal, Montreal, QC, Canada; ^4^West Central Montreal CIUSSS, SHERPA University Institute, Montreal, QC, Canada; ^5^InterActions Centre de recherche et de partage des savoirs, CIUSSS du Nord-de-l'île-de-Montréal, Montreal, QC, Canada

**Keywords:** COVID-19, early childhood, loneliness, migrant mothers, social isolation

## Abstract

**Background:**

Migrant women with young children, including asylum seekers and refugees, have multiple vulnerability factors that put them at increased risk of social isolation and loneliness, which are associated with negative health outcomes. This study explored the experiences of social isolation and loneliness among migrant mothers with children aged 0–5 years as well as their perceptions on possible health impacts.

**Methods:**

A qualitative descriptive study was conducted at La Maison Bleue, a non-profit organization providing perinatal health and social services to vulnerable women in Montreal, Canada. Recruitment and data collection occurred concurrently during the COVID-19 pandemic, between November and December 2020. Eleven women participated in individual semi-structured interviews and provided socio-demographic information. Interview data were thematically analyzed.

**Results:**

Migrant women in this study described social isolation as the loss of family support and of their familiar social/cultural networks, and loneliness as the feelings of aloneness that stemmed from being a mother in a new country with limited support. Multiple factors contributed to women's and children's social isolation and loneliness, including migration status, socioeconomic circumstances, language barriers, and being a single mother. Women expressed that the COVID-19 pandemic exacerbated pre-existing experiences of social isolation and loneliness. Mothers' experiences affected their emotional and mental health, while for children, it reduced their social opportunities outside the home, especially if not attending childcare. However, the extent to which mothers' experiences of social isolation and loneliness influenced the health and development of their children, was less clear.

**Conclusion:**

Migrant mothers' experiences of social isolation and loneliness are intricately linked to their status as migrants and mothers. Going forward, it is critical to better document pandemic and post-pandemic consequences of social isolation and loneliness on young children of migrant families. Supportive interventions for migrant mothers and their young children should not only target social isolation but should also consider mothers' feelings of loneliness and foster social connectedness and belongingness. To address social isolation and loneliness, interventions at the individual, community and policy levels are needed.

## Introduction

The province of Quebec (Canada) admitted 255,966 migrants between 2014 and 2018, with 50.6% being women, most of whom were of childbearing age (15–45 years old), and settling in Montreal (1). Social isolation and loneliness are increasingly recognized as major public health issues, particularly among migrants (2). Social isolation is defined as the objective lack of social contacts and is often measured in terms of social network size, network diversity or frequency of contacts (2). In contrast, loneliness is a subjective experience and refers to feelings of disconnectedness or sadness and absence of meaningful relationships, which occurs when there is a gap between one's desired and actual social experiences (2). Research has found that childbearing migrant women commonly report social isolation and a lack of social support (3, 4). Studies on the topic have mainly focused on women's experiences during pregnancy and the postpartum period and the role of social support as a protective factor toward preventing or limiting social isolation (5). Fewer studies, however, have explicitly explored migrant mothers' experiences of social isolation into the early childhood period, or examined women's perceptions of the health impacts, for themselves, or for their children. There has also been very little attention given to migrant women's experiences of loneliness, especially in the context of the COVID-19 pandemic.

Migrant mothers experience social isolation and loneliness for various reasons. Key factors that may contribute to, or exacerbate social isolation and loneliness, include family separation, language barriers, past traumas, having a precarious immigration status, and unfamiliarity with the host country's culture (6–11). Compounded with multiple vulnerability factors and challenges, the effects of social isolation and loneliness may be even more detrimental for asylum seekers and refugees (3). Existing studies show that social isolation and a lack of support among migrant women are associated with stress, depression and postpartum depression (3, 9–15). An absence of or a small social network has also been shown to contribute to low self-esteem and self-confidence, and a reduced sense of parental competence (6, 13, 16, 17).

Social isolation may also affect the mental and physical health of children, either directly or indirectly, via its impact on maternal mental health. Children aged five and younger are particularly vulnerable since they are highly dependent on the immediate family for care and early stimulations (18–20). Early childhood is a time when children experience rapid physical, developmental and emotional growth, and thus the consequences of social isolation during this period can have long-lasting effects (20).

During the COVID-19 pandemic, efforts to contain the pandemic have forced people to stay at home and limit their social contacts, and thus intensified social isolation and loneliness and their effects, especially for more vulnerable communities. Isolation through confinement and quarantine in the context of COVID-19 have been associated with negative psychological outcomes including stress, depression and post-traumatic stress disorders (21, 22). Moreover, ethnic and racial minorities have been disproportionally affected by the pandemic due to pre-existing social and economic inequalities (i.e., poverty, discrimination, poor access to healthcare) (23). Families with young children may be particularly impacted. Concern about the health and wellbeing of their children can heighten fear and uncertainty among parents. Financial losses may be more distressing, as parents worry about how they will continue to provide for the family. Stress may also be greater due to difficulties in trying to balance work and/or family life responsibilities with child care, especially with reduced access to their usual coping mechanisms and support networks. The psychological effects can have a significant toll and can affect parenting skills. Children are also sensitive to their environments and the emotional state of their parents. Confinement can also reduce children's participation in activities that can be positive for growth and development.

Documenting the experiences of loneliness and social isolation in migrant mothers with young children, as well as the perceived impacts on health, is critical for the development of appropriate interventions for this population. Therefore, this study aimed to answer the following questions:

1. What are the experiences of social isolation and loneliness among migrant mothers of children aged five and younger?
2. What are the perceptions of migrant mothers regarding how experiences of social isolation and loneliness may influence their health and the health of their children?

### Study Setting

This study took place at La Maison Bleue (LMB), a Montreal-based non-profit organization that provides perinatal health and social services to vulnerable mothers during pregnancy up to age five (24). Families followed at LMB have numerous vulnerability factors, including economic instability, low education levels, single parenthood, a history of violence or trauma, and social isolation (25). Based on a recent report, 84% of families followed at LMB identified social isolation, emotional fragility and mental health issues as their main challenges; a significant proportion of these families are migrants, including asylum seekers and refugees (24). LMB's interdisciplinary care team, comprised of social workers, nurses, doctors, midwives, and psycho-educators, provides health and social services that aim to break isolation, foster positive parenting experiences, support child development and promote overall family wellbeing (24).

Since the COVID-19 pandemic was declared a public health emergency in March 2020, the Quebec provincial government imposed varying restrictions according to the evolution of the pandemic. These have included the closure of schools, childcare (except for essential workers) and non-essential services and activities, the banning of indoor gatherings, and limitations on travel (26). Moreover, during all of 2020 many community organizations stopped offering services or re-organized their services so that they could be delivered remotely. Recruitment and data collection for this study took place in November and December 2020 at the start of the second wave of the pandemic. As a result, more stringent restrictions to social gatherings were in place in Montreal and only limited in-person activities were permitted during this period.

## Methods

### Study Design

This study was developed in partnership between McGill University, the University of Montreal and LMB. ML conducted the project as part of her master's degree in nursing and was responsible for developing the protocol, recruiting participants, and collecting and analyzing the data. AVH and LM were the supervisors and SP was the on-site LMB resource person; all three provided guidance throughout the development and execution of the research. The research was conducted at two LMB sites, which are located in ethno-culturally diverse neighborhoods with a high proportion of low-income families; one neighborhood is known for having the largest number of migrants (from all regions of the world) and visible minorities in the city of Montreal (27), while the other neighborhood has a high percentage of migrants from South Asia (Bangladesh, India and Pakistan) (28). A qualitative-descriptive design was used, which is a methodology that involves producing detailed descriptions of phenomena to better understand the perspectives and experiences of people involved in the phenomenon of interest, namely for the purpose of informing clinical practice (29). The study received ethical approval in November 2020 before the commencement of recruitment and data collection.

### Recruitment

Prior to recruitment, ML spent 2 months doing clinical training at LMB, which provided opportunities to become familiarized with LMB's care approach and their clientele. The LMB staff facilitated recruitment by referring and introducing ML to potential participants. Women with any immigration status (refugee, asylum-seeker, non-status, immigrant/permanent resident, or temporary resident/student), and aged 18 years and older were invited to participate if they had arrived in Canada within the last 10 years and were able to communicate in English or French (French is the official language in Quebec). ML was responsible for explaining the study, confirming eligibility and obtaining consent. LMB staff referred 25 women, of which 11 accepted to participate; 10 women refused and four did not respond. Women reported a lack of time and privacy concerns (despite explaining that the research team was obligated to maintain confidentiality) as reasons for refusal of participation.

### Data Collection and Analysis

Individual semi-structured interviews, lasting between 60–90 min, were conducted in French (*n* = 6) and English (*n* = 5). Four mothers had in-person interviews, which were conducted in a private office at or the other of the LMB sites; five mothers were interviewed by phone, and two were interviewed using Zoom Pro. Data were collected using a socio-demographic questionnaire (used in previous research by the senior author) and an interview guide (available as Supplementary Material). The latter was developed by ML, LM, and AVH based on the research objectives. LMB staff reviewed and provided feedback on the study data collection tools before the start of the study.

The socio-demographic questionnaire was administered at the beginning of each interview. The interview topics included participants' immigration and resettlement experiences, their sources of social support, their perceptions related to their health and their children's health, their worries and challenges as parents, and their future goals. ML met regularly with AVH and LM throughout the data collection phase in order to discuss initial impressions of the data, and also strategies for enhancing interview techniques for subsequent interviews. All interviews were audio-recorded and then intelligently transcribed by a professional transcriber. All transcriptions were verified by ML to ensure accuracy. ML also kept general field notes throughout the study, including observations during interviews, and personal reflections and impressions (30, 31).

Data collection and analysis were done concurrently. Data were thematically analyzed using a five-step method to examine the perspectives of different participants and to highlight similarities and differences by sorting and collating codes into categories and generating themes (32). This approach is methodical and rigorous, but also flexible (30, 32). ML coded each transcript using the comment feature in Word. The first two transcripts were also independently coded by AVH and LM, and all three met to discuss and reach consensus on the codes. All codes and categories were then compiled into a table in Word. The codes were then grouped into categories using Excel, and codes and categories were further analyzed by grouping similar codes and categories together to generate themes; no differences by language or region/country of origin were noted. Initially, 237 codes were identified which were then collapsed into 48 codes. Codes were further condensed and regrouped to generate 4 themes, 8 categories and 17 codes (Table 1). The final themes were confirmed through an iterative process of analysis and exchange between the authors.

**Table 1:** Summary of the codes, categories and themes identified during the analysis process.

**Codes**	**Categories**	**Themes**
Collectivist vs. individualistic cultures	Challenges	Social and cultural environments change
Family/partner separation		
Language barriers		
Transnational communication	Protective factors	
Community organization support		
Increased responsibilities	Negative elements	Duality of motherhood
Decreased personal time		
Limited access to affordable childcare		
New purpose in life	Positive elements	
Source of happiness and companionship		
Protect children from maternal challenges	Mother's façade	Mothers' perceptions of children's health
Mothers decreased social contacts	Socially isolated	
Limited opportunities for children to socialize		
Fear	Impact on health	COVID-19 pandemic
Anxiety		
Distress		
Fewer social interactions	Impact on opportunities to socialize	
Remote services and activities		

## Results

### The Participants

The participants' characteristics are reported in Table 2. The participants' ages ranged from 18–39 years old. The women originated from six different countries in Asia and Africa. Six participants self-reported as being an asylum seeker or having a refugee status. All participants considered themselves as “fluent” or “speaking well” in French or English. However, there were two participants with whom communication was more challenging during the interview.

**Table 2:** Socio-demographic characteristics of participants (*n* = 11) at La Maison Bleue (Montreal, Canada), collected between November and December 2020.

**Variables**	**Number**	**%**
Mothers' age		
18-29 years	7	63.6
30-39 years	4	36.4
Country of birth		
India	4	27.3
Democratic Republic of Congo	3	9.1
Benin	1	9.1
Sierra Leone	1	9.1
Mauritania	1	9.1
Sudan	1	9.1
Self-reported immigration status		
Asylum seeker	5	45.5
Permanent resident	2	18.2
Student/Temporary resident	2	18.2
Refugee	1	9.1
Canadian citizen	1	9.1
Years in Montreal/Canada		
< 1 year	1	9.1
2 - 5 years	8	72.7
> 5 years	2	18.2
Highest completed education level		
Secondary and below	1	9.1
Post-secondary and above	10	90.9
Living with		
Alone	3	27.3
Husband/partner	7	63.6
Friends	1	9.1
Number of children (all ages)		
1	4	36.4
2	3	27.3
3 or more	4	36.4
Employment status		
Unemployed and not looking for employment	4	36.4
Unemployed and looking for employment	4	36.4
Part-time employment and student	2	18.2
Full-time employment	1	9.1
Annual income		
$5,000-$19,999	2	18.2
$20,000-$49,999	2	18.2
$50,000-$75,000	1	9.1
Don't know	6	54.4

### Themes

Four themes were identified (see Table 1). The first two reflect the experiences of social isolation and loneliness of migrant mothers and their perceptions of how these experiences may have affected their health: 1) New social and cultural environments following migration: challenges and protective factors; and 2) Duality of motherhood: amplification of social isolation and loneliness and a source of happiness and companionship. The third theme focuses on “Mothers' perceptions of their children's health”, and the fourth theme describes “The COVID-19 pandemic: an exacerbating factor” for social isolation and loneliness for both migrant mothers and their children.

#### New Social and Cultural Environments Following Migration: Challenges & Protective Factors

Key challenges reported by participants included family separation and adaptation to a more individualist culture, as well as language barriers, which resulted in less social support and a lack of meaningful social connections. All eleven participants came to Canada without their immediate or extended families, whom they described as important sources of support and assistance for the care of their children. The physical separation from family and the resulting lack of help led to the mothers feeling a greater sense of burden, stress, and ultimately, loneliness. Ten participants directly expressed feeling “alone” and having to take care of *everything* by themselves. For example, P4 said, “we feel it [loneliness] when we need our family or help”.

Three participants spoke specifically about being separated from their partners, while two others shared that they had no significant partner in their life. These women expressed fear and anxiety due to being single mothers and worried about not having anyone close by who could care for their children should something happen to them. P8 described this as her greatest concern, “When I walk on the street, I imagine if like the car hit me or something happens to me, what will happen to my kids?”

Three women expressed how relationships with others in Canada are different from their home country. In Canada, they often must plan social visits and make appointments for services in advance unlike in their home country where they could see family and friends, depend on their neighbors, and access services without having to schedule encounters ahead of time. They explained that always having to plan and make appointments restricted their access to support. They also felt that people in Canada are generally less available to help. One participant stated, “No one has the time here… Someone can help you for one or two days, but more than that… everyone has their own worries… it's not the same in [my home country]… we help each other within the community” (P4). These differences also seemed to make it more difficult to create meaningful and close relationships, thus adding to participants' feelings of sadness and aloneness. As described by one participant, P5 said, “When I feel really lonely, it bothers me; it makes me sad... I didn't have chances to make friends in Canada... Now, if I need someone to talk to or need help, I can't find anyone on the phone”.

Language barriers further complicated making social connections and accessing support. For example, P11 described how her first 6 months in Canada were difficult because she was not able to connect with neighbors and ask for help: “all neighbor here, people… not too much understand and not help… here all neighbors also speak French. No speak English so too much difficult”.

One way that women dealt with the lack of close relationships and their feelings of loneliness in their new social and cultural environment, was by maintaining contact with their families abroad. Eight women reported communicating regularly (at least once a week) with their family members, while two spoke with their families daily (one to several times a day). Migrant mothers described this contact as a source of moral and psychological support. P6 said, “when I talk to my family it's less lonely… I feel happy and not alone… And change my mind”. For three mothers, they also received advice on childcare. Transnational communication was also a means for women and their children to maintain closeness when separated from their partner/father and siblings. One mother said (P3), “this allowed them [siblings] to bond with each other, so we don't feel a separation between the two”.

All participants also stated that they had some level of assistance or help locally, either through religious or non-profit organizations, friends, neighbors or social services. LMB, particularly, was regarded as an important source of support: “LMB became my family. If I need someone to talk with, I often talk with the LMB staff.” (P1).

#### Duality of Motherhood: Amplification of Social Isolation and Loneliness and a Source of Happiness and Companionship

All eleven participants stated that caring for their children was a priority and that most of their daily activities revolved around caring for their children and doing household work. Tending to the needs of their young children, combined with the lack of access to childcare or more informal support (due to family separation), made it difficult for mothers to create and build a social network, learn English or French, and find paid work. P1 explained, “I wanted to do my courses [in accounting] quickly, but it's difficult with three children… I want to find childcare for the children and to start working” (P1). Similarly, another woman, an asylum seeker, described how not having childcare meant she could not work, and this left her confined to her home and contributed to her sense of stress and boredom. She also explained that cost was the reason she was not able to access childcare:

If we stay at home all day taking care of children, we can't go to work. Staying at home is too stressful. We don't do anything; we only take care of the children. We stay at home all day, 24 h a day. I would like to send my children to childcare, but childcare for asylum seekers is too expensive (P10).

Two other asylum-seeking mothers also expressed that cost prevented them from using childcare, which in turn meant they could not work or partake in other activities which could help them improve their situation. In contrast, four mothers, who had access to childcare, described it as an invaluable resource. P4 explained, “If we didn't have childcare… It's impossible to work… to study. Childcares are really great support.”

Although motherhood and the associated responsibilities contributed to women's isolation, their children also offered them a sense of purpose. Four participants stated not feeling lonely because their children kept them busy. For example, P9 described how she felt before becoming a mother vs. after: “before I was feeling lonely because I was alone. Now I have a family and I am very busy, my schedule, so I don't have time to be lonely”. Ten mothers described how their children were a source of happiness and companionship and thus helped them cope with their situation. For example, P5 said, “Loneliness makes me sad, but then… I have my child. He is 4 years old, but I can talk with him like he is 10 years old. He makes me laugh so much. He is like a friend.”

#### Mothers' Perceptions of Their Children's Health

All participants thought that their children were generally happy and healthy, which they associated with good physical health (e.g., eating well, having no illness), expressions of happiness (e.g., smiling and playfulness), having their needs satisfied (e.g., toys, food) and being loved and cared for. When asked about the health of their children, P9 answered, “she plays, she like, enjoy in the playing and she took feed at the time I feed her… she always gives a smiley face whenever I look at her… they are doing regular check-up and she is fine”. Generally, mothers did not seem to think that their challenges and experiences of social isolation and loneliness were affecting their children because they felt that their children were either too young to understand the situation or because the mothers hid their negative feelings. One woman, however, did say that her child had responded negatively to her stress: “in some situations when I feel stressed, she [her daughter] also gets irritated” (P5). And another mother also described how her child being separated from his father was affecting him: “he feels sad… when he sees his friend go with the father… he keeps asking why my father he is not with us” (P8). Six other participants suggested that experiences of social isolation and loneliness *could* have negative impacts on their children, which is why they made a point to put on a “façade” and hide their feelings to protect their children. P3 described, “I always try to hide [that I'm tired or stressed]… I'm my children's hope here. All their support. And if they feel their support is getting weak, it's easy for them to also get weak”.

Based on the discussions with the mothers, and observations made during the interviews, it seems that young children may have few opportunities to socialize. Mothers' descriptions of their daily and weekly routines with their children indicated that their children tended to have little contact with people outside of home, especially if they were not attending childcare or participating in activities. Eight participants also mentioned that the weather affected their decisions to go outside, which further contributed to keeping their children at home and limiting their activities. For example, P10 stated, “now [in November] I can't [do activities outside]… It's very cold. I can't go out with my children”. P8 explicitly described how delays in accessing childcare and keeping her child at home have had a consequence on her child's language development: “I think my son if since he come, he found a daycare, he should be at this age speaking very well. Because keeping him at home, I'm not free all the time to teach him”. In contrast, mothers whose children had interactions with other children, observed positive outcomes on their children, as described by P5: “He has friends. When he comes back from childcare, he talks about the activities [he did]… he's really starting to express himself, to talk”.

#### The COVID-19 Pandemic: An Exacerbating Factor

Although participants experienced social isolation and loneliness before the COVID-19 pandemic, these experiences were exacerbated by measures put in place to prevent the spread of the virus. Ten participants mentioned that the pandemic has been a challenging period for themselves and/or for their children. It has had an impact on their mood, increased their anxiety and fear, and worsened their situation regarding access to social support and opportunities for self-development and employment.

Four participants reported that physical distancing measures affected their mood or the mood of their children. For example, P6 explained how she could no longer participate in group activities organized by community organizations, and this has left her feeling down and disconnected: “after COVID is very lonely. Not happy. No go out, and always home, it's very boring”. Similarly, P8 shared, in a frustrated tone, “now it's not easy to meet friends… COVID is destroying everything. Yeah, I think my life is not easy because of the COVID… isolation of the 3 months [of lockdown] was tough”.

Four participants expressed fear of contracting the virus and the consequences this might have on their health and the health of their families, and their ability to care for their children:

I have a baby and it's very tough for me to go outside… Because if there's any problem related to COVID, it would also affect my baby and my family… Now we are having fear in mind whenever we are going outside with the baby or alone. (P9)

For the single mothers, the circumstances of the pandemic amplified their challenges and fears. One mother shared:

When COVID started, I was very scared to take my kids with me to have the groceries. And I was like what to do… I don't want to be sick and I'm the only one who is taking care of the kids. (P8)

With the COVID-19 pandemic, participants reported that they limited social interactions and refrained participating in recreational and other activities that could help them secure employment and expand their social network. For example, two participants were no longer attending French classes either because they were canceled or because they feared contracting COVID-19. While some women were able to somewhat break their isolation through online activities, and some even found this option quite advantageous due to its flexibility, it was not an acceptable alternative for all. P8 explained, “because they do the activities online, so I feel not comfortable to join this group… I like to meet people in person”. Four participants also talked about how the restrictions have delayed the initiation of activities for their children. For example, P5 said:

I wanted to register him so that he can do sports… but everything is closed… it's better when [children] start at a young age. I want him to be open; that he's not limited to just childcare and home… that he creates a network of friends… that he strives”.

Lastly, four participants mentioned that the pandemic has had an impact on the support they usually receive through LMB. Before the pandemic, LMB had an open-door policy where women were welcome to drop by at any time. For many women, this approach aligns with what they are used to in their home country. All eleven participants described LMB as an important resource and space where they could socialize, acquire new skills, and discuss their children and childcare: “There were activities at LMB... It felt good to go out and talk with other mothers, to exchange. We can learn things.” (P3). However, due to the pandemic, LMB had to adapt their services and the open-door policy was no longer possible. Some of the women reported feeling that LMB had become less accessible: “With the pandemic, people need to keep a distance. For example, we could go to LMB when we wanted, but it's not the case now. We speak on the phone... the support is different” (P3).

## Discussion

Migrant women in this study described social isolation as the loss of family support and of their familiar social/cultural networks, and loneliness as the feelings of aloneness that stemmed from being a mother in a new country with limited support. Migration status, socioeconomic circumstances, language barriers, and being a single mother, were key factors that shaped these experiences. While women described child-rearing as demanding, their children were also a source of happiness and provided a sense of purpose. Women's experiences of social isolation and loneliness affected their emotional and mental wellbeing, while for children, it reduced their social opportunities outside the home, especially if not attending childcare. While the impacts of social isolation on children could not be elucidated, there were some suggestions that children may be adversely affected. The COVID-19 pandemic also exacerbated the families' situations and experiences.

### Social Isolation and Loneliness in the Context of Migration and Motherhood

Previous research on the experiences of migrant women during pregnancy and motherhood, shows that women feel isolated, lonely and depressed due to the lack of practical assistance from their extended family, especially during the first year when they are still recovering from childbirth and adjusting to their role as mothers (33–35). Migrant women also feel a strong sense of loss due to the absence of support to help maintain traditional practices (33). Our findings align with this earlier work, in that women in our study also described social isolation as being “cut-off” from the support networks they had in their home countries, and as being consumed by motherhood due to a lack of support. Women also described loneliness as feeling a sense of loss and longing for their home country. However, women in our study further elaborated and explained that social isolation was also due to a lack of opportunity, capacity and available time to create social connections, and that this was rooted in their difficulties in accessing resources and services (e.g., childcare), their lack of employment and their financial hardship. Moreover, women felt lonely because the types of support, when available, were not what they were used to, and did not align with their values, thus making it difficult to establish meaningful relationships. Loneliness was also expressed as feeling that there was absolutely no one who they could depend on, especially for those who were single mothers; these women worried that if something were to happen to them, their children would be left without anyone to care for them. The mothers also felt a responsibility to shield their children from their stress and fears, and the negative impacts of their situation.

Motherhood was a key element underpinning the women's experiences of social isolation and loneliness. Postpartum and early childhood are known to be periods when mothers may feel isolated and lonely (36–40). Contributing factors include the burden of care and responsibility related to the mothering role, which can result in women feeling overwhelmed, exhausted, and physically, psychologically and socially disconnected from the outside world (2, 40). These emotions are also linked to women's feelings of vulnerability, instability, and alienation that can arise due to the many life changes that are occurring during this time, including shifts in their relationships with their partner, family members and friends (2, 17, 38–40). For migrant women, these experiences are amplified by the resettlement context. Motherhood, especially for first-time mothers, is a time when women need reassurance, advice, and normalization of their experiences. However, for many migrant women, like the women in our study, especially single mothers, refugees and asylum-seekers, the protective support system is lost with migration (8, 10, 41). Migrant parents are also adapting to new cultural and social norms, which can make them doubt their parental ability, and feel anxiety in their role, and/or leave them disappointed due to different expectations and ideals around family life and parenting (17, 38). Language barriers, and having a precarious immigration status, further intensify the situation by reducing access to support (8, 10, 41). Moreover, when migrant mothers are alone to care for their children, this limits time for self-development (e.g., language classes) and employment, which would help them overcome some of the barriers contributing to their isolation and loneliness (37, 42).

The factors and experiences contributing to migrant women's isolation and feelings of loneliness, including a lack of social support, language barriers, economic difficulties, life and childcare stresses, and having an ethnic minority and/or refugee/asylum seeking status, are also known to be associated with maternal mental health disorders in this population (43). In our study, the COVID-19 pandemic heightened migrant mothers' stress and concerns, generated additional barriers to creating social connections and directly affected their emotional wellbeing. Our results, as well as the emerging literature on the COVID-19 pandemic' impacts on wellbeing and mental health, highlight the vulnerability and the potential severe and long-term effects that the pandemic may have for certain groups (21, 44, 45). For migrant women with young children, especially single mothers, the risk for mental health issues, including depression, is high due to the accumulation of risk factors (e.g., caregiving roles, economic insecurity) (44, 46). Moreover, having pre-existing feelings of loneliness has been shown to compound the risk for depressive symptoms during the pandemic among childbearing and postpartum women (46).

### Social Isolation, Loneliness and Children's Health and Wellbeing

Women in this study generally did not feel that their experiences of social isolation and loneliness affected their children because they thought their children were too young. However, through the mothers' descriptions of their daily routine, we observed that the children had few opportunities for socialization outside the home due to their mothers' limited social contacts. Asylum-seeking women also did not have access to childcare because it was not affordable, so this also contributed to children staying home. In another study, asylum seekers also reported challenges in accessing childcare due to affordability (Quebec has a subsidized childcare program but asylum seekers are not eligible) and reduced social networks (47, 48). Restrictions during the pandemic also further limited children's social interactions since many activities were canceled. Goodman et al. (16) showed that social isolation and loneliness among mothers are associated with adverse child outcomes, including higher levels of internalizing and externalizing behavioral problems, general psychopathology, and negative emotional affect. Studies have also shown that exposure to a stressful home environment, including a depressed mother, can have deleterious effects on children's social, emotional, physical, cognitive and language development (19, 49). Not having access to childcare not only affects migrant families' social integration in the country of settlement, but it may also have negative consequences on migrant children's development since the childcare milieu can stimulate all aspects of development, including language development, which is particularly critical for children of migrants (50, 51).

### Transnational Connections

Transnational connections (i.e., relationships with family members abroad) were described as sources of emotional support and advice for childcare, thereby reducing women's stress levels and loneliness. It also allowed couples, as well as children and fathers who were separated, to maintain closeness. Other studies have also shown that maintaining transnational relationships can help migrants cope with isolation and provide a space of belonging, which involves connecting culturally and in their own language (52–55). However, studies have also found that transnational connections can be a source of stress, and having large and diverse social networks (i.e., transnational ties as well as local social ties) are associated with an overall positive sense of wellbeing (56, 57). We noted that although women maintained strong transnational ties, they often only had small, local social networks. The lack of meaningful connections locally also likely reinforced women to turn toward their transnational relationships, especially during the pandemic.

## Implications

The findings of our study suggest that healthcare providers should not only assess for social isolation, but should also inquire about feelings of loneliness and the circumstances (e.g., single motherhood, immigration status, family separation, and/or different cultural norms) that may be contributing to these emotions in order to identify an appropriate response (25, 58). Interventions for migrant mothers with young children, such as home visit programs and parent groups, often aim to provide social support and childcare assistance to break isolation, reduce stress and decrease women's risks for depression; loneliness, however, has been given less attention (10, 11, 13, 14, 59–62). The findings of this study highlight the importance to also address migrant mothers' disappointment and unhappiness with their social connections. Tackling their loneliness requires increasing their sense of belonging, fostering meaningful and positive friendships with people who they can identify with, and helping them construct a network that will provide reassurance and a sense of community (36, 37, 42).

One approach that the women in this study valued and enjoyed was the “open-door policy” offered at LMB, where women could simply drop-in and spend time; this aligned with the collective, community-oriented support that many had in their home country. A pre-pandemic study also showed that LMB played an important role in helping migrant women expand their social networks by connecting them with other mothers, many of whom shared the same ethnic background or country of origin, and/or who shared similar migration trajectories, which provided a foundation upon which women could bond and create a sense of camaraderie (52).

Community and religious organizations were also identified as helpful. Research has shown previously that these organizations can help reduce loneliness by connecting migrants to resources or by directly offering assistance and by creating a sense of belonging (42). In Quebec, community organizations are integrated into the provincial healthcare network to facilitate referrals and optimize continuity of care (63) and in Montreal there are many that specifically aim to support newcomers and young families living in poverty (64). Community agencies and initiatives, as well as faith-based institutions, however, are often dependent on donations and volunteers to operate, and during the pandemic, resources have been stretched, thus limiting their capacity to meet the needs of their communities (65). In-person events and activities have also been limited, and organizations, LMB included, have had to create new ways to deliver their services. To ensure their survival, additional resources are needed to strengthen these community supports.

Breaking social isolation among migrant women and their children also requires addressing the underlying contributing socio-economic factors; in the province of Quebec, this could include improving access to subsidized childcare for asylum seekers and other government services/programs (e.g., family allowances) (66). Improving access to childcare and strengthening municipal family policies (e.g., social housing, recreational and leisure facilities, and community initiatives) for all families, regardless of migration status, are also imperative (67). Early childhood education and socialization are essential as they promote development and may prevent long-term negative outcomes associated with social inequalities (68, 69). For women, childcare and social policies can facilitate economic and personal development through greater access to employment and skill building courses or activities.

The results also highlight the importance of families having access to affordable internet and communication technologies considering the role of these in maintaining social connections. Not all migrant families may have access to internet and communication devices or are eligible for support programs that help gain access to these (57, 70–73). Many families therefore may not be able to communicate with their family abroad or participate in online local support groups, places of worship, educational activities and health and social services; leaving these families more isolated, especially during the pandemic. More programs and broader eligibility criteria are therefore needed to ensure migrant families have equitable access to technology and telecommunication services.

Lastly, the findings underscore the need for the Canadian and Quebec governments to do more to reunite families (in a timely manner) (74). Numerous policy changes implemented during the pandemic (e.g., temporary closure of international borders), and over the years to manage migration flows and to reduce the “burden” on the Canadian health and social systems, have left many migrants, particularly those who are low-income, living in uncertainty for long periods of time, or unable to reunite with their loved ones. This has led to increased anxiety and feelings of powerlessness for migrants (75, 76). Family reunification not only reduces stress, but can also strengthen intergenerational ties and family cohesion, which in turn promotes overall wellbeing (74).

## Future Research

Additional research on the long-term impacts of the COVID-19 pandemic on migrant families with young children is warranted. Studies examining the effects of social isolation and loneliness on young children of migrants via more direct methods, could be beneficial to enhance our understanding of their experiences and risks, and to inform interventions targeting this population. It would also be worthwhile to further develop interventions to address loneliness among migrant families, especially during and post-pandemic.

## Limitations

Although it is known that language barriers are associated with social isolation and loneliness (6, 8), for feasibility reasons, we had to limit our sample to women who could communicate in English or French. The refusal rate was high; only half of the women referred agreed to participate. The sample size was also small and the timeframe for data collection was short, so data saturation might not have been achieved. The use of different modes (zoom, telephone and in-person interviews) for data collection and the limited observational data gathered during virtual/phone interviews, could have affected the information obtained. We were also not able to capture the experiences of social isolation and loneliness pre- to during pandemic in real-time because we only conducted interviews at one time point with each participant. The women followed at LMB may be a select population and so results may not be applicable to other contexts. The sample, however, was diverse; it included women with different immigration statuses and backgrounds as well as newly resettled women and those who had been in Canada for much longer, and so perspectives were varied. The data collection also happened during the start of the second COVID-19 wave in Montreal, and therefore we were able to capture mothers' actual experiences during the pandemic.

## Conclusion

Migrant mothers' experiences of social isolation and loneliness are intricately linked to their status as migrants and mothers. Mothers' experiences affect their emotional and mental health while for their children, it can impact opportunities for interactions outside the home. The COVID-19 pandemic further exacerbated experiences of social isolation and loneliness and its impacts on migrant women and their families. Supportive interventions for migrant mothers and their young children should not only target social isolation but should also address mothers' feelings of loneliness. To prevent social isolation and loneliness, and to promote a sense of belonging and connectedness, interventions at the individual, community and policy levels are needed.

## Data Availability Statement

The datasets presented in this article are not readily available because they contain information that could compromise the participants' privacy. Excerpts of data are available upon request. Requests to access the datasets should be directed to lisa.merry@umontreal.ca.

## Ethics Statement

The study was reviewed and approved by the Research Review Office of the CIUSSS West-Central Montreal (reference number 2021-2265). The participants provided written or verbal informed consent to participate in this study.

## Author Contributions

ML recruited, obtained consent and interviewed the participants, and took the lead in analyzing the data. AVH and LM supervised recruitment and collection and analysis of the data. ML, AVH, and LM contributed to the interpretation of the results and wrote the manuscript. SP facilitated recruitment and data collection, provided expert knowledge on the target population, and reviewed and provided critical feedback on the manuscript. All authors read and approved the final manuscript.

## Funding

This work was supported by the McGill Global Non-Communicable Diseases Program, the McGill Nursing Collaborative for Education and Innovation in Patient and Family-Centered Care, the SHERPA University Institute, and CERDA (the Centre d'expertise sur le bien-être et l'état de santé physique des réfugiés et des demandeurs du CIUSSS du Centre-Ouest-de-l'île-de-Montréal). AVH and LM were supported by the Fonds de la recherche du Québec – Santé (FRQ-S), Junior 1 Research awards. Publication fees were paid by LM's É*tablissement de jeunes chercheurs* grant, FRQ-S.

## Conflict of Interest

The authors declare that the research was conducted in the absence of any commercial or financial relationships that could be construed as a potential conflict of interest.

## Publisher's Note

All claims expressed in this article are solely those of the authors and do not necessarily represent those of their affiliated organizations, or those of the publisher, the editors and the reviewers. Any product that may be evaluated in this article, or claim that may be made by its manufacturer, is not guaranteed or endorsed by the publisher.
