# Supplementary material for: Social Isolation, Loneliness and Health: A Descriptive Study of the Experiences of Migrant Mothers With Young Children (0–5 Years Old) at La Maison Bleue
Source: Front Glob Womens Health. 2022 Jun 24;3:823632. doi: 10.3389/fgwh.2022.823632 (PMC9265247; doi:10.3389/fgwh.2022.823632)
Supplement: Supplementary file 1 [file Data_Sheet_1.PDF]

Semi-structured Individual Interview Guide

Introduction:

Hello, my name is ______. Thank you for agreeing to participate in this study.

The goal of this study is to better understand immigrant mothers' social experiences and what they think and feel about their health and the health of their children. We will ask you about 1) your immigration experience and your experience living here in Quebec 2) your situation at home with your family and your daily life 3) your social support (i.e. friends, family, neighbors) 4) your thoughts about your health and your children's health 5) your worries and challenges as parents and 6) your future plans and goals.

The interview will last between 1 hour to an hour and a half and I will be taking notes during our discussions. Please note, that you can refuse to answer any questions you do not wish to answer and still remain in the study. You can also stop the interview at any time without having to give a reason.

Before the interview, I will also ask you to fill out a questionnaire, which will be audio-recorded.

Do you have any questions before we start?

Complete socio-demographic questionnaire with participant.

Thank you for your answers. We can now proceed with the interview. Do you have any questions before we start?
I will start the audio-recording.

Interview Guide

* We will let the conversation flow naturally and probes will be used accordingly

<u>Current situation and family situation:</u>

1. **How are you doing? How is your family doing?**

<u>Integration experience:</u>

2. **How do you find living in Montreal/Quebec/Canada?**
   Probes:
   2.1. What are the positive points? What are the challenges?
   (using health and social services, housing, language barriers, communication with people, participating in social activities/social groups, etc.)

3. **Please describe what you usually do in a week (work, activities, children, outings, etc.)?**
   Probes:
   3.1. Do you do any activities outside of the home? Please explain/give some examples.
   3.2. Do you feel like you are doing all of the activities that you would like to do? Please explain.
   3.3. Do you have activities that you would like to do but feel that you cannot do or choose not to do? If yes, please explain.

<u>Social isolation/Loneliness:</u>

4. **Can you describe the support you get from others?** (friends, family and other contacts here in Montreal as well as those back home in your country of origin)
   Probes:
   4.1. Do you feel that you have the support you need? To care for your children?
   4.2. Do you feel your support has changed since coming to Canada? Please explain

5. **Do you ever feel lonely sometimes? If yes, please give some examples of moments where you have felt lonely and describe what this was like for you?**
   Probes:
   5.1. What do you do when you feel lonely? / What do you do to feel less lonely?
      5.1.1. How many people could you call if you needed someone to talk to?
      5.1.2. If you needed help, do you have family or friends (here or in your home country) who could help you?
      5.1.3. Do you feel close to the people around you? Please explain.
   5.2. What would make you feel less lonely here in Canada?
      If she doesn't report loneliness:
      5.2.1. What do you think would help (migrant) mothers of young children like you who feel lonely?

Mother's Health:

6. **How does having social contacts (or not having social contacts) (i.e. friends, neighbors, co-workers, etc.) impact you?** (health, well-being)

7. **Do you think your difficulties or loneliness has an impact on your health or well-being? If yes, how?** (Ask if the person has previously spoken about loneliness or problems of integration and social isolation).

Children:

I would now like to ask you some questions about your children.

8. **How are your children? How is their health?**
   Probes:
   8.1. Do you feel that your children are happy and healthy? Please explain.
   8.2. Do you have any fears or concerns about their health? Please explain.

9. **How did your children react to the challenges or difficulties that you have experienced in Montreal/Canada?**
   Probes:
   9.1. Challenges/difficulties with social relations
   9.2. Do you think your difficulties or loneliness has any influence on them (well-being/health)? If yes, how? (Ask if the person has previously spoken about loneliness or problems of integration and social isolation).

Future:

10. How do you see yourself and your child/children in the future?

Do you have any comments or questions that you would like to share or ask me before we end this interview?

Thank you for taking the time to answer my questions. The interview is now over.
