# Supplementary material for: Social Isolation, Loneliness and Health: A Descriptive Study of the Experiences of Migrant Mothers With Young Children (0–5 Years Old) at La Maison Bleue
Source: Front Glob Womens Health. 2022 Jun 24;3:823632. doi: 10.3389/fgwh.2022.823632 (PMC9265247; doi:10.3389/fgwh.2022.823632)
Supplement: Supplementary file 2 [file Data_Sheet_2.PDF]

**Socio-Demographic Questionnaire**

**PARTICIPANT CODE: _____________**

**LA MAISON BLEUE SITE:** ☐ Parc-Extension ☐ Côte-des-Neiges

1. **What is your age?**

   - 18 – 29 years old
   - 30 – 39 years old
   - 40 – 49 years old
   - 50 years or older
   - Chose not to answer

2. **What is your marital status?**

   - Married
   - Consensual union (unmarried partners)
   - Widowed
   - Separated
   - Divorced
   - Single
   - Chose not to answer

3. **Who do you live with? Check all that apply.**

   - Husband/Wife
   - A female or male partner (for unmarried partners)
   - Your Mother/Father
   - Your Brothers/Sisters
   - Your Partner's Mother/Father
   - Friend(s)
     or
   - I live alone (pregnant and/or just with children)

4. **How many children do you have? How many live with you? (Check all that apply)**

   - ___________ (number of children) ________ (number of children living with you)
   - Currently pregnant

5. **When (what year) were your children born? Where were they born and were they followed at La Maison Bleue at the time of their birth?**

   - _________ (year), _______ (country), followed at La Maison Bleue ☐ Yes ☐ No
   - _________ (year), _______ (country), followed at La Maison Bleue ☐ Yes ☐ No
   - _________ (year), _______ (country), followed at La Maison Bleue ☐ Yes ☐ No
   - _________ (year), _______ (country), followed at La Maison Bleue ☐ Yes ☐ No
   - _________ (year), _______ (country), followed at La Maison Bleue ☐ Yes ☐ No
   - _________ (year), _______ (country), followed at La Maison Bleue ☐ Yes ☐ No

   - **No children born yet**

*The next set of questions ask information about your immigration history. We are interested in this information because we want to learn more about the experiences of different migrants to Canada. Any answers you provide will remain confidential and answering these questions will not affect your immigration application if you are in the process of applying for refugee status, permanent residency, or citizenship.*

6. **What is your country of birth? ___________________________**

7. **How long have you lived in Canada?**

   ___________ (months) _____________ (years)

8. **How long have you lived in Montreal?**

   ___________ (months) _____________ (years)

9. **What is your current immigration status?**

   - Immigrant (permanent/landed status)
   - Refugee
   - Refugee Claimant/Asylum-Seeker
   - Temporary worker/Live-in caregiver
   - Temporary resident
   - Student
   - Visitor
   - No status
   - Undocumented
   - Citizen
   - Other (please specify): ___________________________
   - Chose not to answer

10. **How long have you had this status?**

   ___________ (months) ____________ (years)

**11. Did you ever have refugee status?**

- o Yes
- o No
- o Don’t know

*The next set of questions are more general questions about you.*

**12. What is the highest level of education that you have completed?**

- o Primary school
- o Secondary diploma
- o Postsecondary diploma (e.g trade school, college, university)
- o Graduate diploma (Master’s, Doctoral)
- o None
- o Chose not to answer

**13. What is your current work situation?**

- o Working full time
- o Working part-time
- o Not working and not looking for work
- o Unemployed and looking for work
- o Currently in school
- o Chose not to answer

**14. If you are working, what is your current job? ___________________________________**

**15. What are your sources of income (Check all that apply)?**

- o Employment/Job
- o Partner’s employment/job
- o Help from relatives
- o Social assistance (unemployment, child support, disability)
- o Chose not to answer

**16. What is approximately your yearly household income?**

- o Less than $5,000
- o $5,000 - $19,999
- o $20,000 - $49,999
- o $50,000 - $ 75,000
- o More than $75,000
- o Don’t know
- o Chose not to answer

**17. How many people live in your household and live off of the income above?**

- 2 people and less
- 3-4 people
- 5-6 people
- 7 people and more

**18. What is your mother tongue/first language? ___________________________________**

**19. How well do you know French?**

| | Fluent | Well | With Difficulty | Not at all |
|---|---|---|---|---|
| **Speak** | | | | |
| **Read** | | | | |
| **Write** | | | | |
| **Understand** | | | | |

**20. How well do you know English?**

| | Fluent | Well | With Difficulty | Not at all |
|---|---|---|---|---|
| **Speak** | | | | |
| **Read** | | | | |
| **Write** | | | | |
| **Understand** | | | | |
